# Separation of Water/Oil Emulsions by an Electrospun Copolyamide Mat Covered with a 2D Ti_3_C_2_T_x_ MXene

**DOI:** 10.3390/ma13143171

**Published:** 2020-07-16

**Authors:** AbdolAli Moghaddasi, Patrik Sobolčiak, Anton Popelka, Igor Krupa

**Affiliations:** 1 Biological and Environmental Science Department, Qatar University, P.O. Box 2713, Doha, Qatar; am1404948@student.qu.edu.qa; 2 Center for Advanced Materials, Qatar University, P.O. Box 2713, Doha, Qatar; patrik@qu.edu.qa (P.S.); anton.popelka@qu.edu.qa (A.P.)

**Keywords:** MXene, copolyamide, membrane, water purification, vegetable oil

## Abstract

**Purpose:** Copolyamide 6,10 (coPA) electrospun mats were covered with multilayered (ML) and single-layered (SL) MXene (Ti_3_C_2_T_x_) as a membrane for the separation of water/vegetable oil emulsions. **Methods:** Prepared membranes were characterized by atomic force microscopy (AFM), profilometry, the contact angle measurements of various liquids in air, and the underwater contact angle of vegetable oil. The separation efficiency was evaluated by measuring the UV transmittance of stock solutions compared to the UV transmittance of the filtrate. **Results:** The MXene coating onto coPA mats led to changes in the permeability, hydrophilicity, and roughness of the membranes and enhanced the separation efficiency of the water/vegetable oil emulsions containing 10, 100, and 1000 ppm of sunflower vegetable oil. It was found that membranes were highly oleophobic (>124°) under water, unlike in air, where the membranes showed high oleophobicity (<5°). The separation efficiency of water/oil emulsions for both types of covered membranes reached over 99%, with a surface coverage of 3.2 mg/cm^2^ Ti_3_C_2_T_x_ (for ML-Ti_3_C_2_T_x_) and 2.9 mg/cm^2^ (for SL-Ti_3_C_2_T_x_). **Conclusions:** The separation efficiency was greater than 98% for membranes covered with 2.65 mg/cm^2^ of ML-Ti_3_C_2_T_x_, whereas the separation efficiency for membranes containing 1.89 and 0.77 mg/cm^2^ was less than 90% for all studied emulsion concentrations.

## 1. Introduction

The continuous improvement of people’s living standards is associated with intensive catering industry development and has resulted in a large amount of restaurant wastewater. Restaurant and household wastewater consists of many animal and vegetable oils and various colloids, detergents, proteins, plant fibers, and inorganic salts, which make it difficult to purify. The untreated direct discharge of catering wastewater can reach more than 100 million tons each year [1]; however, in some regions, untreated restaurant wastewater is not allowed to be discharged directly into the municipal sewerage network [2], or no infrastructure has been made yet [3]. Generally, restaurant wastewater treatment includes flotation, sand filtration, coagulation, membrane bioreactor (MBR), and biological turntable, along with an aerobic biological sludge process [4]. However, these conventional techniques suffer from limitations such as a low separation efficiency, high energy cost, and non-applicability to oil/water emulsion systems [4,5]. On the other hand, membranes are widely used for various types of separation processes. Microfiltration membranes have been studied continuously in oil/water separation, but they are mostly used in the petrochemical industry and are seldom used in the catering industry.

The separation of oil/water mixtures and emulsions can be achieved by combining a suitable surface structure and composition design [6]. 

The electrospinning of various polymeric materials was found to be a versatile tool to fabricate nanofibrous materials with controllable compositions and structures [7,8] by tailoring remarkable characteristics such as high surface-to-volume ratio and multiporosity, along with chemical, physical, and mechanical functions provided by incorporating other components [9,10]. On the other hand, some limitations and challenges such as pore structure controllability, shrinkage, and the distortion of electrospun structure need to be taken into consideration [7].

Many polymers have been employed for the preparation of nanofibrous mats suitable for water/oil separation. Among them, polyamides are favorable due to their mechanical properties and thermal stability [11]. Moreover, it has been demonstrated that an improvement on the separation properties of electrospun mats can be obtained through their modification by nanomaterials introduced onto their surface or directly into the fibrous structure. Many various types of nanofillers have been studied to fulfill the application requirements.

MXenes, which are 2D transition metals [12], have been recently prepared by Gogotsi and Barsoum groups [13] by the chemical etching of “A” from a MAX phase, where M is a transition metal, A is a group IIIA or IVA element, and X is C or N. Due to their extraordinary physical and chemical features, such as electrical conductivity and hydrophilicity, MXene have been used for electrochemical supercapacitors [14], catalytic promoters [15], absorbents for heavy metal ions [16], materials for the photodegradation of dyes [17], and anti-biofouling membranes [18,19].

For instance, Du et al. [20] studied the demulsification of acidic oil-in-water emulsions driven by chitosan-loaded Ti_3_C_2_T_x_ powder. They succeeded with a demulsification efficiency of up to approximately 67% with an increased dosage of chitosan loaded Ti_3_C_2_T_x_. 

Zhang et al. described an MXene membrane obtained by depositing Ti_3_C_2_T_x_ MXene 2D nanosheets carbides onto porous polyvinylidene fluoride (PVDF) membranes by vacuum filtration. The as-prepared Ti_3_C_2_T_x_ MXene membrane exhibits excellent underwater superoleophobicity. The MXene membrane separated a series of stable emulsions that were even emulsified crude oil-in-water mixtures and displayed excellent separation efficiency over 99.4% and a high permeation flux of 887 L/m^2^ h bar [21].

Li et al. proposed an ultra-thin 2D titanium carbide MXene membrane (about 30 nm) supported on porous polyethersulfone as substrate with high performance and excellent stability for oil-in-water emulsion separation. The high antifouling resistance, promising oil/water separation, and excellent recyclability of the MXene membranes originated from their inherent hydrophilicity, low adhesion of oil droplets, as well as the regular stacking of the 2D lamellar structure that was observed [22].

The preparation and characterization of membranes that are based on an electrospun copolyamide (coPA) as a substrate and a coating of Ti_3_C_2_T_x_ that acts as the active component repelling the vegetable oil is reported in this paper. 

## 2. Materials and Methods 

### 2.1. Materials

CoPA (Vestamelt X1010, EVONIK Industries, Essen, Germany), *n*-propanol >99.5% (Sigma Aldrich, St. Louis, MO, USA), Ti_3_AlC_2_ purity >99% (Y-Carbon, Ltd., Kiev, Ukraine), hydrochloric acid 37% (Sigma Aldrich, St. Louis, MO, USA), lithium fluoride 99.5% (Sigma Aldrich, St. Louis, MO, USA), and vegetable oil (Adams group, St. Louis, MO, USA) were all used in this experiment. Ultra-pure water (prepared by Purification System Direct Q3, Millipore Corporation, France), formamide >99.5% (Sigma Aldrich, St. Louis, MO, USA), and ethyleneglycol >99% (Sigma Aldrich, St. Louis, MO, USA) were used as testing liquids for the contact angle measurements.

### 2.2. Preparation of the Ti_3_C_2_T_x_

Ti_3_C_2_T_x_ was prepared according to a protocol published elsewhere [23]. Briefly, the MAX phase was etched by hydrofluoric acid (HF) produced in situ via lithium flouride (LiF) and hydrochloric acid (HCl) at temperature 40 °C overnight. Then, multilayered Ti_3_C_2_T_x_ (ML-Ti_3_C_2_T_x_) was cleaned by extensive centrifugation until the pH of the supernatant was over 4. Prepared ML-Ti_3_C_2_T_x_ was filtrated through a membrane with pores of 0.3 µm, washed with ethanol, and dried at 40 °C. Single-layered Ti_3_C_2_T_x_ (SL-Ti_3_C_2_T_x_) was prepared by inner probe sonication of the multilayered Ti_3_C_2_T_x_ at amplitude of 40% and cycle 0.5 for 20 min in water. Then, MXene suspension was centrifuged at 2000 rpm for 60 min. Subsequently, supernatant containing SL-Ti_3_C_2_T_x_ was collected and dried. Terminated groups (T_x_) in prepared Ti_3_C_2_T_x_ are based on oxygen, hydroxyl, and fluorine.

### 2.3. Preparation of the coPA Electrospun Mats

The coPA mats were prepared by electrospinning 18 wt % coPA in n-propanol. 

The polymer solution was electrospun on a NanospiderTM laboratory scale machine (Elmerco, Liberec, Czech Republic) with an emitter voltage of +45 kV and a collector voltage of −33 kV. The distance was set to 200 mm. Ambient conditions were controlled and set to a relative humidity of 30% and a temperature of 23 °C. The draining speed of the material was set at 20 mm/min.

### 2.4. Fabrication of the Ti_3_C_2_T_x_-Covered coPA Membrane 

The coPA/Ti_3_C_2_T_x_ membranes were fabricated by a deposition of a 50 mg Ti_3_C_2_T_x_ in 50 mL of deionized water dispersion through a coPA mat fixed between two glass funnels. Then, the membrane with the formed Ti_3_C_2_T_x_ layer on the surface was extensively washed with water to remove unattached Ti_3_C_2_T_x_ particles until the water filtrate did not contain any Ti_3_C_2_T_x_ particles. This step was confirmed by UV spectroscopy. Prepared membranes were dried in a vacuum at 60 °C overnight, and the weight of the deposited Ti_3_C_2_T_x_ particles was calculated by comparing the weight of the dried coPA mat to the dried coPA/Ti_3_C_2_T_x_ membrane after Ti_3_C_2_T_x_ particle deposition.

### 2.5. Characterizations

The surface morphology of the specimens was examined with field emission scanning electron microscopy (FE-SEM, Nova Nano SEM 650, Hitachi, Japan) equipped with an energy-dispersive X-ray spectrometer (EDS). All specimens were sputter-coated with 2 nm of gold before the SEM images were taken. The EDS measurements were performed from three points of the specimens.

The morphology of the Ti_3_C_2_T_x_ particles was investigated using transmission electron microscopy (TEM, JEM-2100Plus, JEOL, Peabody, MA, USA). Powdered Ti_3_C_2_T_x_ was dispersed in distilled water to obtain a 0.05 wt % dispersion. Then, a drop of dispersion was deposited directly on the TEM grid and evaporated under ambient conditions. 

X-ray diffraction (XRD) analysis was performed on a Bruker D8 ADVANCE X-ray diffractometer (Bruker Corp., Billerica, MA, USA) equipped with Cu Kα radiation (λ = 0.154 nm). The scanning range (2θ) was from 5° to 60° at a scan speed of 2°/min. 

UV spectra were recorded with a spectrometer (SEC2000-UV/VIS, ALS, Tokyo, Japan) using a quartz cuvette. The data were obtained at a constant bandpass with a resolution of 2 nm.

The OCA35 optical system (Data Physics, Filderstadt, Germany) was used to measure the wettability of prepared samples by a sessile drop technique. Liquids with a different surface tension (water, formamide, ethylene glycol, vegetable, and diesel oil) were used to investigate the wettability of the prepared coPA membranes. A volume of 1 µL of each testing liquid was used to eliminate the gravitational effects when studying the contact angle. A representative value of the contact angle was obtained from 10 separate readings. The angle between the solid/liquid and liquid/vapor interface was referred to as the contact angle. 

Atomic force microscopy (AFM) was used to further elucidate the 2D morphology and 3D topography of the prepared membranes surface using an MFP-3D AFM microscope (Asylum Research, Santa Barbara, CA, USA) equipped with a silicon probe (Al reflex-coated Veeco model—OLTESPA, Olympus; spring constant: 2 N/m, resonant frequency: 70 kHz). Measurements were performed under ambient conditions using the Standard Topography AC air (tapping mode in air). An AFM head scanner with a vertically adjacent Si cantilever was applied in the sample resonant frequency of the free-oscillating cantilever and set as the driving frequency. An inverted optical microscope (Olympus, Tokyo, Japan) with a custom Asylum Research Base plate mounted on top to support the AFM head was used for obtaining images of emulsions and permeates.

### 2.6. Separation of Water from Oil-In-Water/Oil Emulsions

**Scheme 1:**
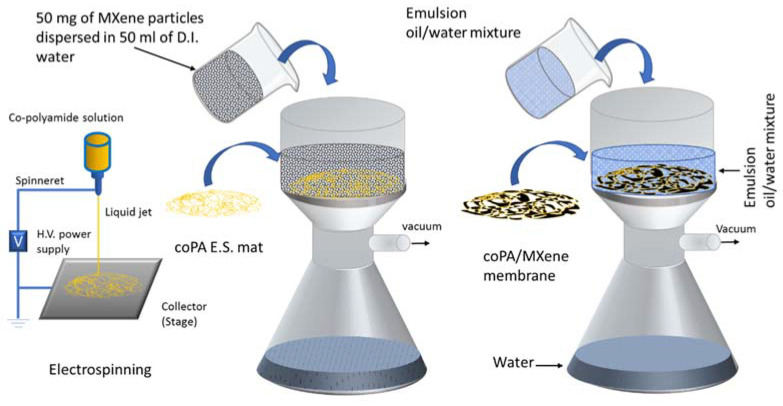
Experimental setup of filtration experiments.

For oil/water separation, the neat coPa mat and membranes covered by ML- and SL-Ti_3_C_2_T_x_ were fixed between two glass funnels with an effective separation area of 9.61 cm^2^ as illustrated at Scheme 1. An emulsion of oil/water with an oil concentration of 10, 100, or 1000 ppm was probe sonicated at a frequency of 40% and cycle of 0.5 for 15 min while cooling in an ice bath. This step was followed by 24 h of stirring at RT to ensure good dispersion. Subsequently, 50 mL of the emulsion was poured into the upper test glass vessel and through the as-prepared membrane. In the case of experiments with various amounts of ML-Ti_3_C_2_T_x_, 200 mL was used as a single run. The performed separation was driven by using a vacuum pump at constant pressure of 0.5 bar. The filtrate was collected, and the efficiency of separation was analyzed by UV spectroscopy (SEC2000-UV/VIS, ALS, Tokyo, Japan) according to Equation (1) with a Suprasil quartz cuvette (10 mm) from Heraeus Quarzglas GmbH. The data were obtained at a constant bandpass with a resolution of less than 2 nm.

Calibration fit was generated to obtain the concentration of oil in water from UV spectroscopy data, and the efficiency of separation was calculated by using Equation (1):

(1) $$efficiency=1-\left(\frac{c\left(filtrate\right)}{c\left(stock\;solution\right)}\right)*100\;\left(\%\right)$$

where efficiency was the efficiency of separation in %, c(filtrate) was the ppm concentration of oil in the filtrate, and c(stock solution) was the ppm concentration of oil in the stock solution [9].

The flux through the membranes was calculated according to Equation (2):

(2) $$J=\frac{V}{A\cdot t}\;\left(L/m^{2}\cdot h\right)$$

where *J* is the flux, *V* is the permeate volume (L), *A* is the effective test area (m^2^), and *t* is time (h).

## 3. Results

### 3.1. Preparation of Ti_3_C_2_T_x_

**Figure 1:**
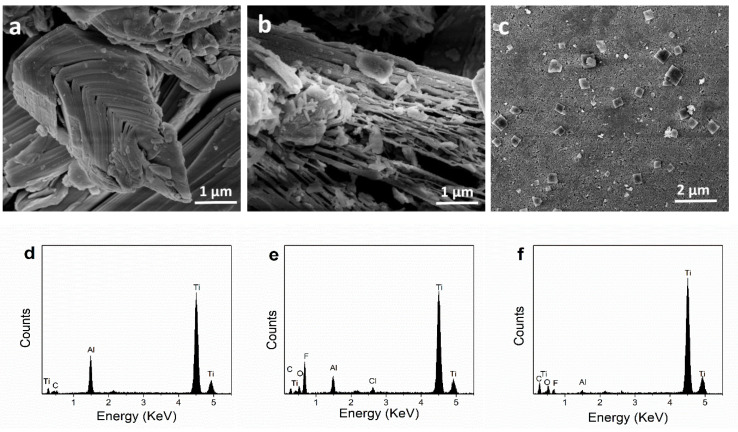
Scanning electron microscopy (SEM) images of: (**a**) Ti_3_AlC_2_, (**b**) multilayered (ML)-Ti_3_C_2_T_x_, and (**c**) delaminated single-layered (SL)-Ti_3_C_2_T_x_. Energy-dispersive X-ray spectrometer (EDS) spectra of (**d**) Ti_3_AlC_2_, (**e**) multilayered ML-Ti_3_C_2_T_x_, and (**f**) delaminated SL-Ti_3_C_2_T_x._

Ti_3_C_2_T_x_ was prepared from Ti_3_AlC_2_ (Figure 1a) by the etching of Al via an in situ-produced HF (through a reaction of LiF and HCl). This process caused the exfoliation of the Ti_3_C_2_T_x_ layers (Figure 1b) and the formation of the so-called multilayer-exfoliated Ti_3_C_2_T_x_. Then, the extensive sonication of multilayered Ti_3_C_2_T_x_ led to the delamination of multilayered-Ti_3_C_2_T_x,_ and single- or few layered-Ti_3_C_2_T_x_ that was terminated by fluorine, oxygen, and hydroxyl groups prepared with dimensions of Ti_3_C_2_T_x_ under 1 µm (Figure 1c).

An advantage of using LiF/HCl was that both etching and intercalation were achieved in the exfoliation process, which significantly simplified the delamination of Ti_3_C_2_T_x_ [23].

The EDS analysis of Ti_3_AlC_2_, ML-Ti_3_C_2_T_x_, and SL-Ti_3_C_2_T_x_ is shown in Figure 1d–f. The strong signal of characteristic peaks of Ti, C, and Al confirming the Ti_3_AlC_2_ structure are presented in Figure 1d. The ML-Ti_3_C_2_T_x_ image showed decreasing intensity for the Al peak which was caused due to the etching of Al from the Ti_3_AlC_2_ structure. Moreover, new peaks that belong to fluorine and oxygen-terminating groups and were introduced to the structure of exfoliated Ti_3_C_2_T_x_ are observed. Minor peaks belonging to Cl, as impurities, are observed due to etching with LiF/HCl [24]. Prepared SL-Ti_3_C_2_T_x_ contained main Ti, C, O, and F peaks and almost negligible Al and Cl peaks, indicating the high purity of prepared SL-Ti_3_C_2_T_x_.

**Figure 2:**
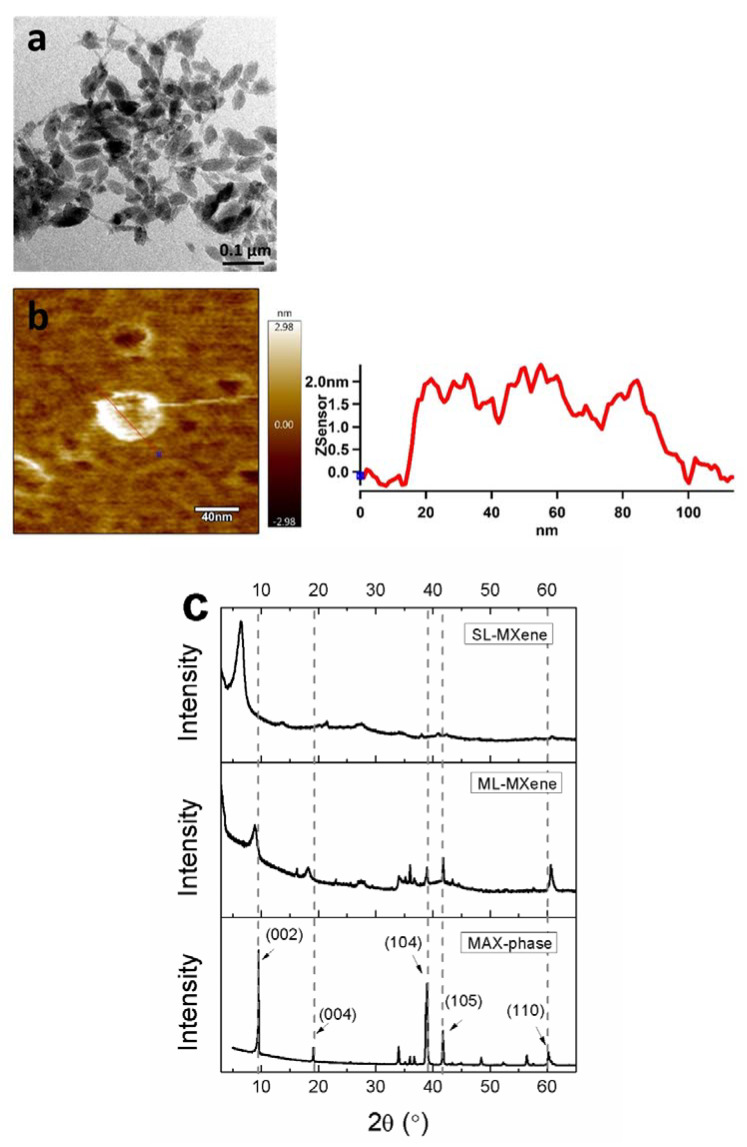
(**a**) transmission electron microscopy (TEM), (**b**) atomic force microscopy (AFM) of the Ti_3_C_2_T_x_ nanosheets, and (**c**) X-ray diffraction (XRD) of Ti_3_AlC_2_ and ML and SL Ti_3_C_2_T_x._

Figure 2a shows the TEM images of Ti_3_C_2_T_x_ nanosheets after delamination with dimensions of approximately 50 × 30 nm and a thickness of 2 nm for the nanosheets with confirmation by the AFM measurements (Figure 2b). 

XRD analysis confirmed the successful removal of Al between the layers of MXene of Ti_3_AlC_2_ (Figure 2c). The characteristic (002) peak of Ti_3_AlC_2_ at 9.5° broadens and shifts to a lower value for both ML and SL-Ti_3_C_2_T_x_, indicating the removal of Al and subsequent structural expansion due to substitution of Al with –F and –OH/O terminating groups, resulting in a larger d-spacing. In addition, the non-basal plane peaks of Ti_3_AlC_2_, most notably the peak at 39°, significantly lost intensity for ML-Ti_3_C_2_T_x_ and even disappeared for SL-Ti_3_C_2_T_x_. The (001) peaks, such as the (002), (004), and also (110) peak, broadened, decreased in intensity, and shifted compared to their locations before etching, which was mainly caused by removing Al from the Ti_3_AlC_2_ [25]. 

### 3.2. Preparation and Characterization of Electrospun Mats and Membranes

**Figure 3:**
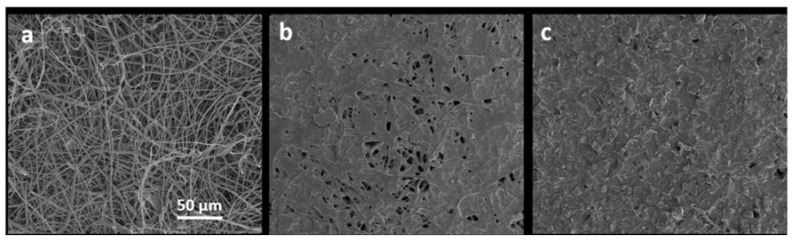
SEM and profile images of (**a**) the copolyamide (coPA) electrospun mat, (**b**) the coPA mat covered by SL-Ti_3_C_2_T_x_, and (**c**) the coPA mat covered by ML-Ti_3_C_2_T_x._

The coPA mats were prepared from *n*-propanol solution by a Nanospider™ electrospinning device, which allowed for easy scale-up of the mat fabrication process. The prepared coPA mats were composed of nanofibers with a uniform distribution. The thickness of the fibers was approximately 800 nm, as confirmed by SEM measurements (Figure 3a). Figure 3b,c shows coPA mats covered by the SL-Ti_3_C_2_T_x_ or ML-Ti_3_C_2_T_x_, respectively. The SL-Ti_3_C_2_T membrane shows few open pores, which could affect the flux of the membrane. On the other hand, the ML-Ti_3_C_2_T membrane exhibited more uniform MXene layer deposition, which is also supported by AFM analysis.

**Figure 4:**
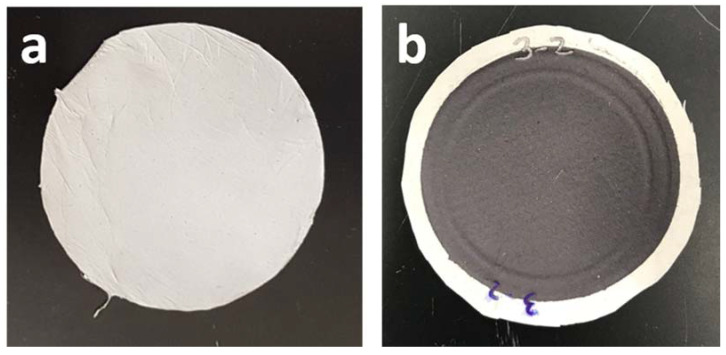
(**a**) neat coPA mat and (**b**) coPA/SL-Ti_3_C_2_T_x_ membrane.

In the next step, electrospun coPA mats were covered with ML- or SL-Ti_3_C_2_T_x_ by vacuum filtration. Due to the high porosity of the coPA membranes, some of the Ti_3_C_2_T_x_ particles penetrated into the 3D structure of the coPA mat, and some of them passed through the mat. The neat coPA mat and the covered coPA membrane are displayed in Figure 4. The Ti_3_C_2_T_x_ particles were distributed homogenously onto the coPA membrane, as shown in Figure 4b.

**Figure 5:**
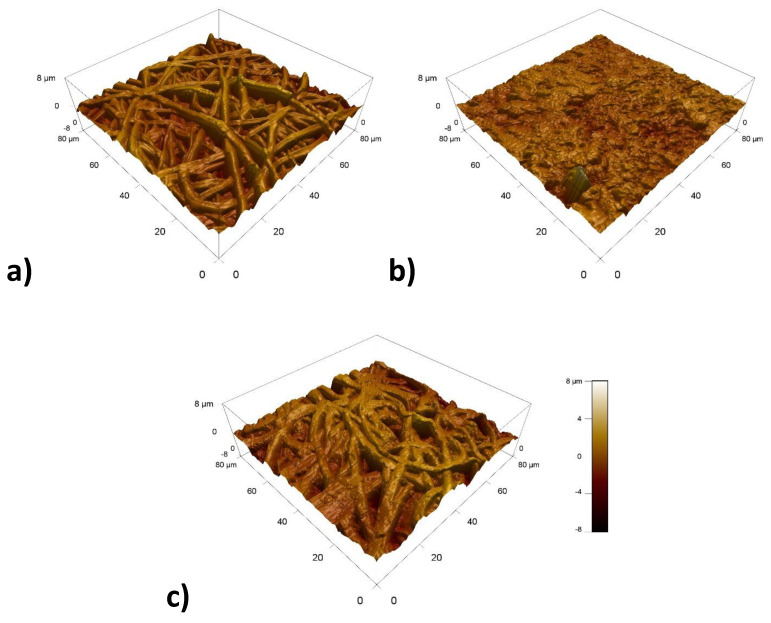
AFM images of (**a**) the neat coPA mat, (**b**) the coPA membrane covered by ML-Ti_3_C_2_T_x_ and (**c**) the coPA membrane covered by SL-Ti_3_C_2_T_x._

The topography of the membrane surfaces was studied using the AFM technique. Figure 5a shows the AFM images of the neat coPA mat. The fibers created a 3D net with a random orientation and high porosity. The membrane covered with ML-Ti_3_C_2_T_x_ (Figure 5b) showed a continuous layer of Ti_3_C_2_T_x_ particles on the coPA mat, which made the surface of the membrane dense. This topography might inhibit the passing through of oil droplets and together with the oleophobicity of the Ti_3_C_2_T_x_ layer (CA) was predicted to be efficient for application in oil removal. 

The membrane covered by SL-Ti_3_C_2_T_x_ is shown in Figure 5c. The SL-Ti_3_C_2_T_x_-covered coPA mats contained particular fibers on the surface of the mat that retained the primary fiber structure. In contrast, for the ML-Ti_3_C_2_T_x_, the fibers formed a thick layer on the surface of the membrane and most likely caused the significant differences in the particle sizes of ML- and SL-Ti_3_C_2_T_x_.

The gravimetrically measured density coverage of the ML-Ti_3_C_2_T_x_ covered onto the membrane was 3.2 mg/cm^2^, whereas the density coverage of the SL-Ti_3_C_2_T_x_ covered onto the membrane was 2.9 mg/cm^2^.

### 3.3. Contact Angle Measurements

**Figure 6:**
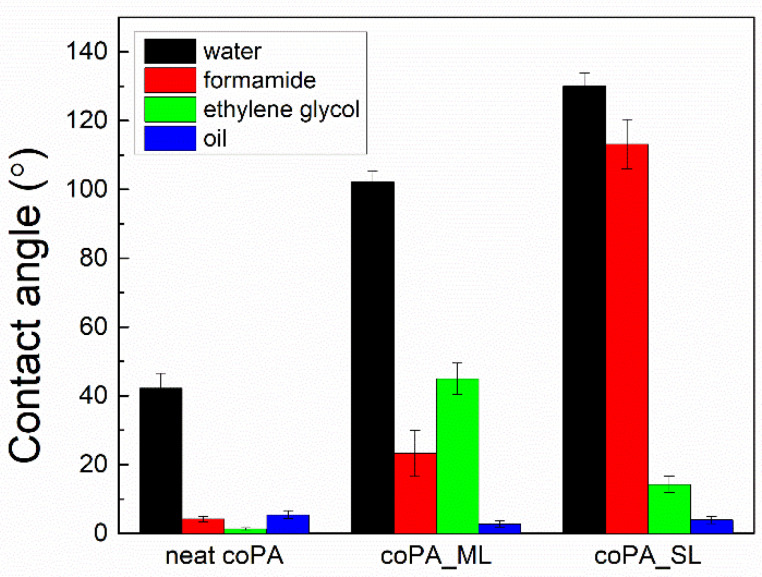
Contact angles for different solvents on the coPA, coPA/ML-Ti_3_C_2_T_x_, and coPA/SL-Ti_3_C_2_T_x_ membranes.

The contact angles of water, formamide, ethylene glycol, and oil with coPA, coPA/ML-Ti_3_C_2_T_x_, and coPA/SL-Ti_3_C_2_T_x_ are shown in Figure 6. The coPA, thanks to the –CO and –NH functional groups on its structure, was considered a hydrophilic polymer. The contact angles of water, formamide, ethylene glycol, and oil on neat coPA mats were 42°, 4°, 1°, and 8°, respectively. The contact angles of water, formamide, ethylene glycol, and oil on the coPA/ML-Ti_3_C_2_T_x_ membrane were 100°, 20°, 45°, and 3°, respectively, and the contact angles of coPA/SL-Ti_3_C_2_T_x_ were 130°, 111°, 15°, and 5° for water, formamide, ethylene glycol, and oil, respectively. 

It needs to be mentioned that the contact angle is strongly dependent on the roughness and overall morphology of the specimen surface. Moreover, the contact angle for various liquids is different if measured in air and under water. The results mentioned above may indicate that membranes are not suitable for water/oil separation (low repulsion of oily droplets due to a low contact angle) and are not suitable for a sufficient flow rate through a membrane (high contact angle for water). However, the experimental facts were not in line with those expectations based only on simple contact angle measurements in air. For the targeted application of oil/water separation, the contact angle measurement of oil under water was more relevant.

**Figure 7:**
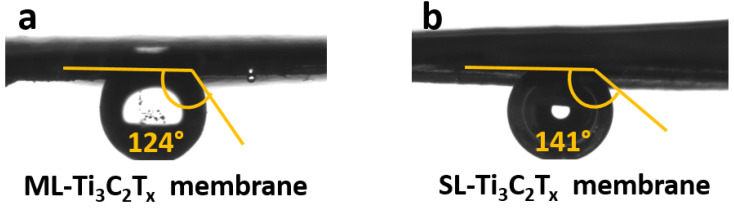
Underwater contact angle of vegetable oil on (**a**) ML-Ti_3_C_2_T_x_ and (**b**) SL-Ti_3_C_2_T_x_ membranes.

To mimic real conditions during oil/water separation, the underwater contact angle of the vegetable oil of the ML- and SL-Ti_3_C_2_T_x_ membranes was measured (Figure 7). The average contact angles of oil on the ML-Ti_3_C_2_T_x_ and SL-Ti_3_C_2_T_x_ membranes were 124° ± 2° and 141 ± 3°, respectively, which was unlike the results from the contact angles of oil performed in air and proved the oleophobicity of Ti_3_C_2_T_x_ layers. This feature of Ti_3_C_2_T_x_ was essential for oil/water separation. 

### 3.4. Water/Oil Separation

**Figure 8:**
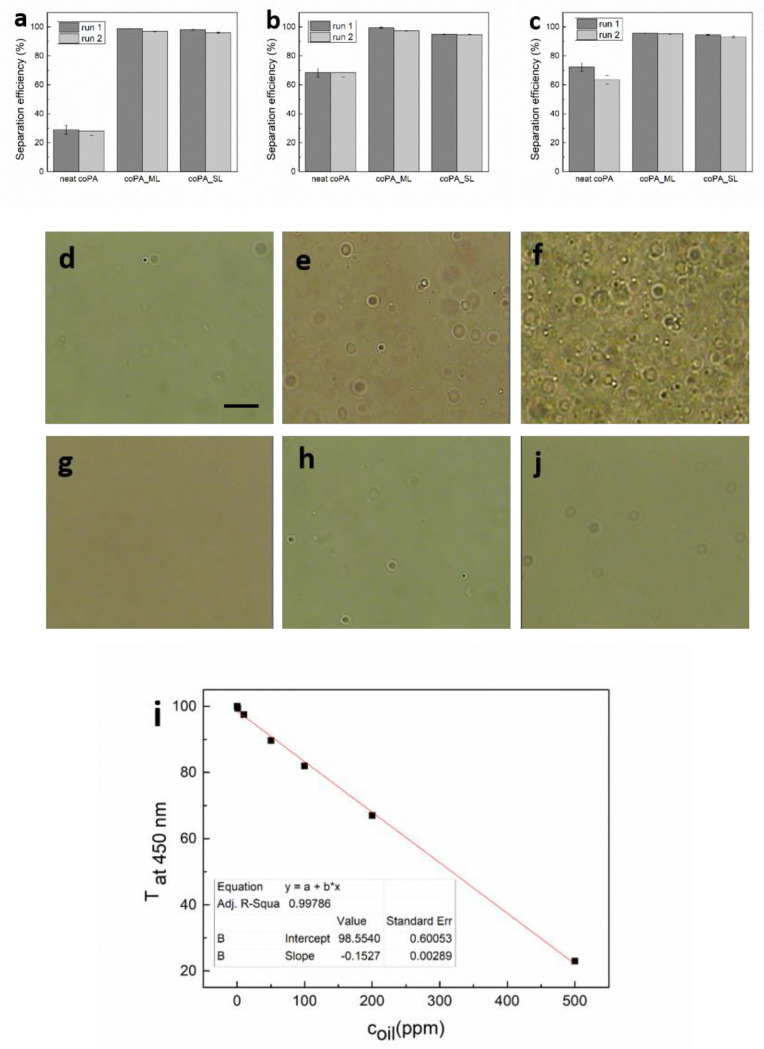
UV spectroscopy of filtrates obtained after separation of (**a**) 10, (**b**) 100, and (**c**) 1000 ppm vegetable oil in water through a neat coPA mat and the coPA covered with ML- and SL-Ti_3_C_2_T_x_ at constant pressure of 0.5 bar. Optical microscopy images of (**d**) 10 ppm emulsion, (**e**) 100 ppm emulsion, (**f**) 1000 ppm emulsion, (**g**) permeate of 10 ppm emulsion, (**h**) permeate of 100 ppm emulsion, and (**j**) permeate of 1000 ppm emulsion. Bar is 1 μm. (**i**) Calibration fit for various concentration of vegetable oil emulsions at transmittance of λ = 450 nm.

The prepared electrospun coPA mats as well as the membranes modified by ML-Ti_3_C_2_T_x_ or SL-Ti_3_C_2_T_x_ were tested for oil separation in oil/water emulsions. Vegetable oil concentrations of 10, 100, and 1000 ppm were tested by filtering through the membranes. Figure 8 summarizes the separation efficiency for the neat coPa, coPA/ML-Ti_3_C_2_T_x_, and coPA/SL-Ti_3_C_2_T_x_ membranes. The separation efficiency for 10 ppm vegetable oil (Figure 8a) through the neat coPA mat showed only 18% separation efficiency (concentration of oil in permeate was 18 ppm), while the coPA/ML-Ti_3_C_2_T_x_ membrane exhibited a separation efficiency of 99.5% (concentration of oil in permeate was 0.05 ppm) and the separation efficiency increased up to 99.8% (concentration of oil in permeate was 0.02 ppm) for the coPA/SL-Ti_3_C_2_T_x_. Figure 8b shows the efficiencies for 100 ppm vegetable oil/water separation. The neat coPA mats exhibited separation efficiencies up to 68% (concentration of oil in permeate was 32 ppm). The separation efficiency increased to 98% (concentration of oil in permeate was 2 ppm) for the coPA/ML-Ti_3_C_2_T_x_ membrane and to 99% (concentration of oil in permeate 1 ppm) for the coPA/SL-Ti_3_C_2_T_x_ membrane. Similar separation efficiency was found for 1000 ppm vegetable oil (Figure 8c). Two runs for each separation were performed, and it was found that a slight decrease in the separation efficiency was observed when comparing the first and second runs. The size of oil droplets in emulsion for 10, 100, and 1000 ppm (Figure 8d–f) emulsions as well as their permeates (Figure 8g,h,j) was examined by optical microscopy. The average size of droplets was 410 nm ± 60 nm, 440 nm ± 55 nm, and 490 ± 210 nm for 10 ppm, 100 ppm, and 1000 ppm emulsions, respectively. As the emulsion concentration increases, the number of oil droplets also increases. On the other hand, permeates contain only few oil droplets due to the successful filtration of oil emulsion through SL-Ti_3_C_2_T_x_ and ML-Ti_3_C_2_T_x_. Figure 8i shows a calibration fit for the vegetable oil emulsion for concentrations from 0 to 500 ppm. 

**Figure 9:**
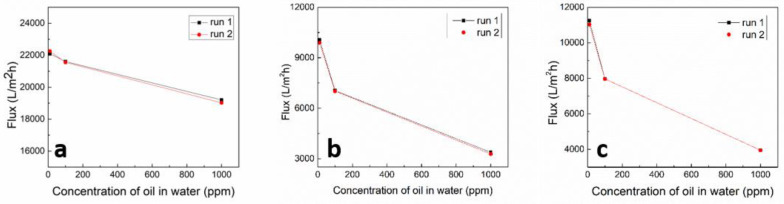
Flux of emulsions through the membranes of the (**a**) neat coPA, (**b**) coPA/ML-Ti_3_C_2_T_x_, and (**c**) coPA SL-Ti_3_C_2_T_x_ at a constant pressure of 0.5 bar.

Figure 9a shows flux oil in water for neat coPA mats. Due to the structure of the membrane having high porosity, very high fluxes have been observed for all investigated concentrations of oil in water, from 19,200 up to 22,100 L/m^2^h at a constant pressure of 0.5 bar. Notably, the flux of the emulsions through the membranes decreased with an increased oil concentration. Figure 9b shows the flux of oil in the water emulsion with 10, 100, and 1000 ppm of oil through the coPA/ML-Ti_3_C_2_T_x_ at a constant pressure of 0.5 bar. The flux for 10 ppm was approximately 10,000 L/m^2^h, which decreased to 7000 L/m^2^h for 100 ppm and then decreased more to approximately 3500 L/m^2^h for 1000 ppm. A similar trend, decreased flux with an increased concentration of oil, was observed for the coPA/SL-Ti_3_C_2_T_x_ (Figure 9c). Our membranes have noticeably higher fluxes retaining the comparable efficiency of separation when comparing with membranes prepared by MXene particles [21,22]. 

It can be concluded that the separation performance of the coPA membranes covered by either ML-Ti_3_C_2_T_x_ or SL-Ti_3_C_2_T_x_ showed a very high separation efficiency, while a high flux was maintained for all emulsion concentrations (10 to 1000 ppm) studied. 

**Figure 10:**
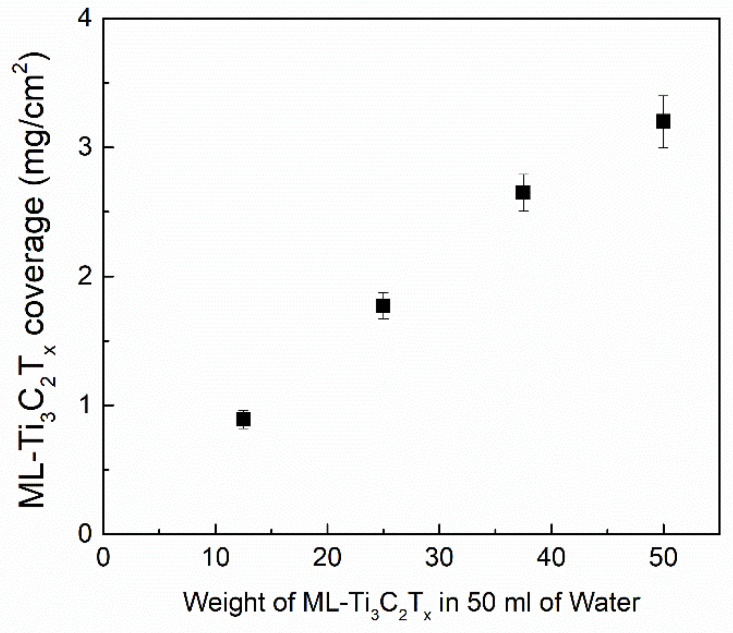
ML-Ti_3_C_2_T_x_ density coverage for the membranes by using various concentrations of the ML-Ti_3_C_2_T_x_ suspension.

In the next step, the content of Ti_3_C_2_T_x_ on the membranes was decreased to study the minimal amount of Ti_3_C_2_T_x_ required while maintaining a high separation efficiency for the membranes. Due to the simplicity of the preparation, ML-Ti_3_C_2_T_x_ was chosen for this study. Three different concentrations of ML-Ti_3_C_2_T_x_ suspension were prepared, which contained 37.5, 25, or 12.5 mg of Ti_3_C_2_T_x_ in 50 mL of water and resulted in Ti_3_C_2_T_x_ density coverage of 2.65, 1.77, and 0.89 mg/cm^2^, respectively (Figure 10).

**Figure 11:**
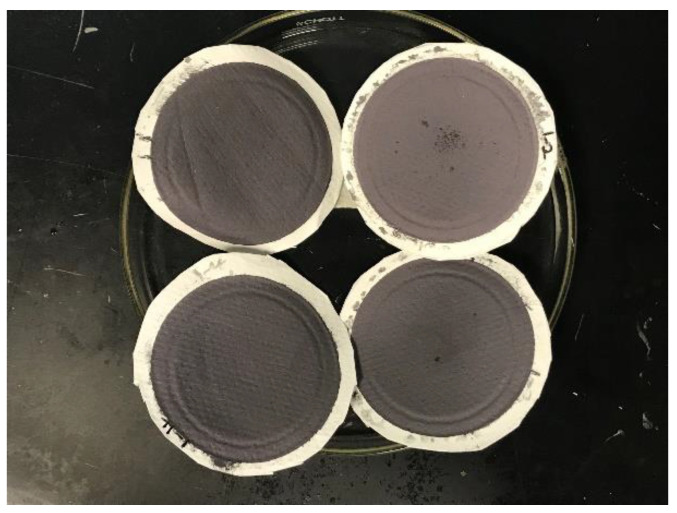
Macroscopic images of membranes covered by various suspension concentrations of ML-Ti_3_C_2_T_x._

The macroscopic images of prepared membranes covered by ML-Ti_3_C_2_T_x_ are shown in Figure 11. All assigned membranes had a good, uniform ML-Ti_3_C_2_T_x_ coverage distribution without any visible defects.

**Figure 12:**
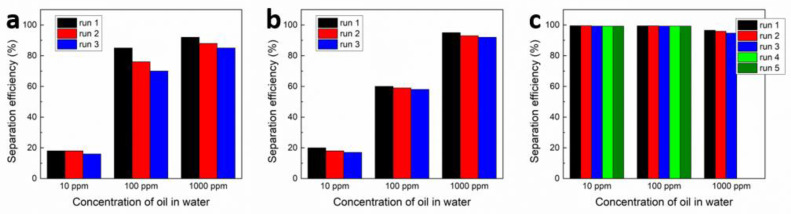
Separation efficiency of the coPA membranes with an ML-Ti_3_C_2_T_x_ density coverage of (**a**) 0.89, (**b**) 1.77, and (**c**) 2.65 mg/cm^2^.

Figure 12 summarizes the separation efficiency of ML-coPA membranes with various Ti_3_C_2_T_x_ coverage for 10, 100, and 1000 ppm oil in water. Each concentration of the oil mixture was filtered with multiple runs to study the repeatability and reusability of the membranes.

Figure 12a shows the separation efficiency of coPA covered by 0.89 mg/cm^2^. A separation efficiency below 20% (concentration of oil in permeate was 8 ppm) was observed for 10 ppm oil in water. Then, the separation of 100 ppm oil in water exhibited an 84% separation efficiency (concentration of oil in permeate was 16 ppm) in the first run, and a slight decrease in the separation efficiency occurred for the second and third runs with a 70% separation efficiency (concentration of oil in permeate was 30 ppm) during the third run. The best results were achieved for the separation of 1000 ppm with a separation efficiency of 91% (concentration of oil in permeate was 90 ppm) for the first run and decreasing to 85% (concentration of oil in permeate was 150 ppm) for the third run of filtration.

The coPA membrane with a higher Ti_3_C_2_T_x_ coverage (Figure 12b), 1.77 mg/cm^2^, showed similar separation performance as the previously studied membrane; it had separation efficiencies below 20% (concentration of oil in permeate was above 8 ppm) for 10 ppm, approximately 80% (equal to concentration of oil in permeate was 20 ppm) for 100 ppm, and 92% (concentration of oil in permeate was 80 ppm) for 1000 ppm oil in water. All separation efficiencies exhibited declining trends for the second and third filtration runs. 

Overall, both membranes showed low separation efficiency, especially for 10 and 100 ppm oil in water, which was caused by the insufficient Ti_3_C_2_T_x_ coverage of the membranes.

The coPA membrane prepared last had a Ti_3_C_2_T_x_ coverage of 2.65 mg/cm^2^ and exhibited high separation efficiency for all studied oil/water mixtures (Figure 12c). The separation efficiency was studied with up to five filtration runs to determine the long-term performance of the membranes. The separation efficiency for 10 ppm oil in water was over 99% (concentration of oil in permeate was 0.1 ppm) for each run. Similar results were observed for the separation of 100 ppm oil in water. In the case of the 1000 ppm separation of oil in water, the separation efficiency of the first run was 98% (concentration of oil in permeate was 20 ppm) and slightly decreased for the second and third run (95%, concentration of oil in permeate was 50 ppm, for the third run). Two additional runs were not able to be performed due to blocking of the membranes.

## 4. Conclusions

The membranes were fabricated with *n*-propanol to form an electrospun coPA supportive layer with Ti_3_C_2_T_x_ layers deposited on top. The Ti_3_C_2_T_x_ layers acted as an active component that repelled vegetable oil. Two different Ti_3_C_2_T_x_ grades, multilayered and single-layered constituents, were used for deposition onto the nanofibrous coPA mats. Moreover, multilayered Ti_3_C_2_T_x_ was used to study the membrane performance (separation efficiency and flux) based on the influence of various amounts of Ti_3_C_2_T_x_ deposited onto the coPA membranes. The minimum amount of Ti_3_C_2_T_x_ that required the separation efficiency over 90% was 2.65 mg/cm^2^.

Prepared membranes were highly oleophobic under water (>124°), with a high separation efficiency of up to 99.5% and an extraordinarily high flux of over 11,000 L/m^2^h for the SL-Ti_3_C_2_T_x_ thanks to its hydrophilic nature.
